# Use of electroconvulsive therapy in adolescents with schizophrenia in China

**DOI:** 10.1186/s13034-018-0254-z

**Published:** 2018-12-05

**Authors:** Shuai Wang, Chao Yang, Junpu Jia, Yuming Zhou, Yi Zheng

**Affiliations:** 1 Beijing Anding Hospital, Capital Medical University, Ankang Road 5, Xicheng District, Beijing, China; 2 Beijing Institute for Brain Disorders, Beijing, 100001 China; 3 The National Clinical Research Center for Mental Disorders, Beijing, China

## Abstract

**Background:**

Electroconvulsive therapy (ECT) is an effective treatment for psychiatric disorders such as schizophrenia, major depression and bipolar disorder. However, few studies have addressed the use of ECT in adolescents with schizophrenia. The aims of our study were to investigate the frequency of ECT, and its relationship with clinical and demographic correlates among adolescents with schizophrenia in China.

**Methods:**

The study was a retrospective study and conducted in the Child and Adolescent Psychiatry Department of Beijing Anding Hospital, and adolescents with schizophrenia over a period of 10 years (2007–2016) were enrolled. The demographic and clinical data were collected from the electronic chart management system.

**Results:**

A total of 835 patients were included, 411/835 (49.2%) of the adolescent inpatients diagnosed with schizophrenia were in ECT group. There were significant differences in the sex, age, high risk for aggression and suicide, family history of psychiatric disorders and concomitant psychotropic medication (antidepressants and benzodiazepines) between the ECT and non-ECT groups. Multiple logistic regression analysis revealed that ECT use was independently and positively associated with sex, high risk for suicide.

**Conclusions:**

In a major psychiatric center in China, the use of ECT was common, and reasons for the high use of ECT for adolescent patients in this hospital should warrant urgent investigations.

## Background

The introduction of electroconvulsive therapy (ECT) for the treatment of serious mental disorders such as schizophrenia, major depression and bipolar disorder, was one of the most impacting revolutions of psychiatry [1, 2]. The frequency of ECT use varies across countries and regions. For instance, ECT use in psychiatric inpatients ranged from 0.01% in Thailand to 1.8% in Hong Kong and 13.4% in India [3–5]. Many researchers believed that ECT use is associated with better symptom relief, lower treatment costs, and shorter hospital stay [6]. ECT use is influenced by a host of legal, social and cultural factors [7–11].

China has a large patient population receiving ECT, a retrospective chart review of 19,982 inpatients aged 18 to 59 years showed that the frequency of ECT use was 66.3% in major depression, 55.2% in schizophrenia, 68.4% in bipolar disorders and 28.6% in other psychiatric disorders [12]. A prospective study of 1364 aged 18 years and older inpatients demonstrated that the percentage of ECT use was 57.0% in schizophrenia, 53.4% in major depression, 57.8% in bipolar disorder and 32.4% in other diagnoses [13]. Zhang et al. examined the frequency of ECT for Chinese Adolescent Psychiatric Patients, and they concluded that the rate of ECT use was 46.5% for patients with schizophrenia, 57.8% for bipolar disorders, 41.8% for major depressive disorder and 23.9% for other diagnoses [14].

Electroconvulsive therapy is a safe and effective treatment in adult patients with schizophrenia, especially when rapid response is needed [15]. In children and adolescents, some studies examining the efficacy of ECT have shown significant benefits in the treatment of schizophrenia [16, 17]. We focused on adolescent patients diagnosed with schizophrenia in China. The objectives of our study were to investigate (1) the frequency of ECT use in adolescents with schizophrenia (2) its demographic and clinical correlates.

## Methods

### Setting and subjects

The study was a retrospective study, approved by the local institutional review board committee. The study was conducted in Beijing Anding Hospital, a tertiary-care academic teaching hospital. This hospital has 800 beds and serves 20 million people from Beijing and other areas, and 50 beds in child and adolescent psychiatry ward, receiving about 500 children and adolescents every year. Since this hospital is the China’s National Clinical Research Center for Mental Disorders, so a considerable proportion of inpatients is treatment-resistant.

Electroconvulsive therapy is mainly provided for inpatients in this hospital. The courses of ECT usually comprise six to twelve sessions for adult under general anesthesia, five times in the first week and then three times per follow week, between 9:00 a.m. and 11:00 a.m. Anesthesia is induced with propofol (1–1.5 mg/kg) accompanied by succinylcholine (0.3–0.7 mg/kg) and oxygenation. Adolescent patients usually receive fewer sessions than adults. A brief pulse wave device with bitemporal electrode placement was used (Spetrum 5000Q ECT machine, MECTA Corp, Lake Oswego, OR). On the first treatment, a pulse width between 0.5 and 1.0 ms was used with the empirical dose titration method, during the ECT course, the pulse was adjusted between 0.25 and 1.0 ms as needed according seizure quality.

### Collection of demographic and clinical factors

The electronic chart management system (ECMS) contains detailed information on diagnoses, risk factors, investigations, prescriptions, hospital referrals, outcomes, and basic demographic information, which was routinely asked by professionals in Beijing Anding Hospital. The ECMS contained data on the risk for suicide and aggression at admission, which was evaluated by a scale designed for all inpatients [12, 13], and the Positive and Negative Syndrome Scale for schizophrenia (PANSS) was evaluated by two psychiatrists who have received the training on the PANSS. PANSS is a 30-item rating scale that aims at assessing the symptom severity of subjects with psychosis. It contains three subscales—positive, negative, and general psychopathology—and a total score. Each subscale and the total score are all evaluated from 1 to 7 according to the severity of the symptoms.

The inclusion criteria were: (1) Inpatients, The length of hospitalization is more than 14 days (2) age, range from 13 to 17 (3) the diagnosis was schizophrenia, which were defined by two psychiatrists, according to the diagnostic criteria of the International Statistical Classification of Diseases and Related Health Problems (ICD-10). if the patient had more than 1 diagnosis, the primary diagnosis was used. Exclusion criteria included the patients with pervasive developmental disorder or neurological disorders. We analyzed data of patients with schizophrenia from July 1, 2007 through December 31, 2016. This work was approved by the Human Research and Ethics Committee of Beijing Anding Hospital. From ECMS, we collected the information including two main aspects: (1) Demographic characteristics: sex, age and the family history of psychiatric disorders. (2) Clinical characteristics: the risk for aggression, the risk for suicide, the length of hospitalization, the number of hospitalizations, the PANSS scores, concomitant psychotropic medication including second-generation antipsychotics (SGAs), first-generation antipsychotics (FGAs), antidepressants, benzodiazepines and mood stabilizers.

### Statistical analysis

The SPSS was used to analyse these data. The comparisons of demographic and clinical variables between ECT and the non-ECT groups were analyzed by using Chi square test for categorical variables, independent samples t-tests and Mann–Whitney U test for continuous variables as appropriate. A binary logistic regression analysis was conducted to assess which factors were significantly associated with ECT group. A stepwise multiple regression analysis was then used to identify independent demographic and clinical correlates of ECT with the “enter” method in the whole sample. In addition, Bonferroni corrections were applied to each test to adjust for multiple testing. The level of significance was set at 0.05 (2 tailed).

## Results

A total of 835 patients including 439 boys (52.6%) and 396 girls (47.4%) were included in our study. The mean age was 15.5 ± 1.44 years, and the length of current hospitalization was 36.7 ± 15.47 days. Altogether 411 patients (49.2%) received ECT. The most frequently prescribed medication were SGAs (97.3%), followed by benzodiazepines (59.4%), antidepressants (22.4%), mood stabilizers (15.1%) and FGAs (6.7%) (Table 1).

**Table 1:** Basic demographic and clinical characteristics of the study sample

	The whole sample (N = 835)		ECT group (N = 411)		Non-ECT group (N = 424)		Statistics		
	N	%	N	%	N	%	X	DF	P
Male	439	52.6	246	59.9	193	45.5	17.2	1	< 0.001
High risk for aggression	246	29.5	174	42.3	72	17	64.6	1	< 0.001
High risk for suicide	77	21.2	124	30.2	53	12.5	39.0	1	< 0.001
Family history	98	11.7	60	14.6	38	9	6.4	1	0.011
SGAs	812	97.3	403	98.1	409	96.5	2.0	1	0.160
FGAs	56	6.7	26	6.3	30	7.1	0.2	1	0.665
Antidepressants	187	22.4	115	28.0	72	17.0	14.5	1	< 0.001
Benzodiazepines	496	59.4	268	65.2	228	53.8	11.3	1	0.001
Mood stabilizers	126	15.1	59	14.4	67	11.1	0.3	1	0.559

	Mean	SD	Mean	SD	Mean	SD	Z		
Age (year)	15.5	1.44	15.9	1.31	15.2	1.48	9.2		0.003
Hospitalizations (n)	1.86	1.12	1.88	1.15	1.83	1.01	0.15		0.696
Length of hospitalization (days)	36.7	15.47	36.4	15.27	37.0	15.67	0.24		0.628

There were significant differences in the sex, age, high risk for aggression and suicide, family history of psychiatric disorders and concomitant psychotropic medication (benzodiazepines and antidepressants) between ECT and non-ECT groups (Table 1). Multiple logistic regression analysis revealed that ECT use was independently and positively associated with sex, high risk for suicide (Table 2).

**Table 2:** Factors independently associated with ECT use (multiple logistic regression analysis)

	P	Odds ratio	95% CI
High risk for aggression	0.636	0.893	0.560–1.426
High risk for suicide	< 0.000	0.374	0.227–0.617
Family history	0.423	1.271	0.707–2.285
Sex	< 0.000	0.552	0.441–0.742

The PANSS scale was used to rate the change in psychopathology, The pre-treatment PANSS refers to the time point at which the treatment was instituted and post-treatment PANSS refers to the time when the patients were discharged from hospital. We assessed the percentage of responders as defined by a 20% or greater reduction in PANSS total scores in relation to their initial evaluation [18], and set 30% for reference. For ECT group, 326 (79.3%) patients had more than 20% reduction in PANSS scores. When the effectiveness was evaluated in terms of more than 30% reduction in PANSS scores, 65% (267 out of 411) scored above this cut off. For non-ECT group, 321 (75.7%) patients had more than 20% reduction in PANSS scores. When the effectiveness was evaluated in terms of more than 30% reduction in PANSS scores, 60.4% (256 out of 424) scored above this cut off.

**Fig. 1:**
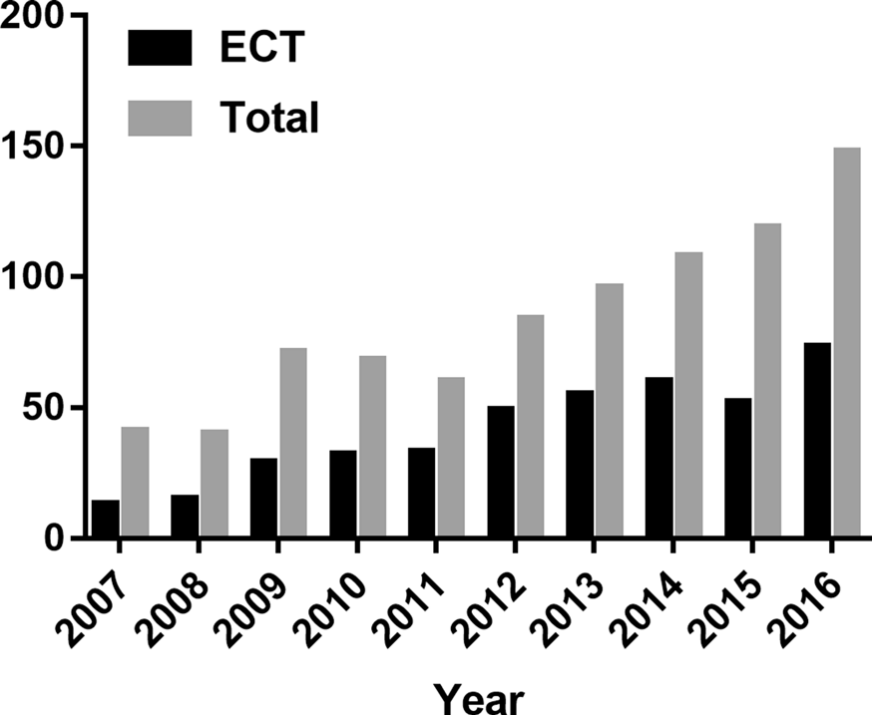
The number of ECT use by year (2007–2016)

Figure 1 showed the proportion of ECT in the whole sample by calendar year. The frequency of ECT use varied between 31.7 and 58.3% during this period. The mean number of sessions was 9.1 ± 3.6 in the whole ECT group.

## Discussion

In this study, we found that 49.2% of adolescent with schizophrenia were prescribed ECT in a tertiary psychiatric hospital in Beijing. The frequency of ECT and its relationship with clinical and demographic characteristics are reported in previous studies, Wang et al. demonstrated that ECT use was associated with higher risk for suicide and aggression, age younger than 30 years, lower risk for fall at time of admission, more prescriptions for mood stabilizers, SGAs, FGAs and antidepressants, less health insurance and major medical conditions, and the ECT use was 55.2% in adults with schizophrenia [12]; a prospective study showed patients receiving ECT had higher education level and younger age of onset, were younger and less likely to be employed, received benzodiazepine, had personal income more than 3000 RMB per month, and the ECT use was 57.0% in adults with schizophrenia [13]; Zhang et al. showed that ECT use was independently and positively associated with older age, high risk for aggression at admission, and the use of FGAs, SGAs, and antidepressants, and the ECT use was 46.5% in adolescents with schizophrenia [14], Consistent with these studies, our study also demonstrated a high rate of ECT use in patients with schizophrenia, and there were significant differences in the high risk for aggression and suicide and concomitant psychotropic medication (benzodiazepines and antidepressants) between non-ECT and ECT groups. A possible reason was that adolescents with schizophrenia who comorbid depression or other conditions are more vulnerable to suffer from the ECT use. This might explain the association between concomitant psychotropic medication and ECT use found in this study. These results demonstrate that ECT use is very common among inpatients in this region, and call for more concern and need for more investigation.

Because of the limited data and the retrospective nature of this study, the reasons for the exceptionally high frequency of ECT in Chinese adolescents with schizophrenia are unclear. Several factors may contribute to our findings. This hospital is The National Clinical Research Center of Mental Disorders that is a tertiary referral center receiving treatment-resistant patients from other hospitals, and these patients are more likely to meet the indications for ECT [12]. Furthermore, treatment-resistant schizophrenia is one of the main indications of ECT in China [19]. Another aspect is, in China, ECT was performed for decades with little controversy without the societal prejudice or pressure to restrict or monitor its use in China [10].

The American Academy of Child and Adolescent Psychiatry (AACAP) has issued a guideline titled “Practice Parameter For Use of Electroconvulsive Therapy With Adolescents which involves three criteria as follows: (1) diagnosis (2) severity of symptoms and (3) lack of treatment response to appropriate psychopharmacological agents accompanied with other appropriate treatment modalities. According to AACAP’s guideline, ECT is mainly recommended for the treatment of MDD, SZ and BD, particularly for these patients with aggressive behavior, suicidal behavior and catatonia. The symptoms of patients may be persistent, severe and significantly disabling, what may cause life-threatening symptoms, such as refusal to drink or eat, florid psychosis, uncontrollable mania and severe suicide intentions [20]. The lack of treatment response when considered at least two adequate trials of correct drugs is also one of the three criteria, and the fulfillment of this criterion may require patient’s observation and thorough medical evaluation. ECT may be considered earlier in cases when psychopharmacological treatment is not effective for the patient, when adolescent is significantly incapacitated, not being able to take medication, or when waiting for response of psychopharmacological treatment may put the patient’s life at risk [21]. However, there are no specific guidelines on the use of ECT for adolescents in China. In general, ECT is mainly recommended for the treatment of SZ, MDD and BD, particularly for patients with aggressive or suicidal behavior and catatonia [22].

The decision to prescribe ECT is largely influenced by the public attitudes toward ECT, efficacy, side effects and costs, clinical traditions, and local treatment guidelines [23–28]. ECT may result in some adverse effects including impairment of memory and new learning, prolonged seizures, risks associated with general anesthesia, and other minor effects. However, some studies do not support this opinion. De la Serna et al. showed no significant differences in change over time in clinical or neuropsychological variables between the ECT group and the non-ECT group at 2-year follow-up. Thus, ECT did not show any negative influence on long-term neuropsychological variables in their sample [29]. In a systematic review of 39 studies, Lima et al. found that ECT use is a highly efficient option for treating several psychiatric disorders in adolescents with few and relatively benign adverse effects [30]. More investigations regarding the adverse effects of ECT are needed, especially in adolescent population.

There were some limitations in our study: (1) The results of this study should be interpreted with caution due to it is a retrospective study. (2) Several relevant variables, such as the dose of psychotropic medications, were not recorded in the ECMS. Therefore, more detailed analyses could not be performed. (3) The study site was a tertiary psychiatric center in China, thus the findings cannot be generalized to other types of facilities and other regions. (4) ECT parameters, such as electrode placement, titration methods, and electric charge were not recorded in the ECMS. Therefore, the findings should be regarded as preliminary and should be confirmed before the firm conclusions could be made. (5) We did not have access to some factors such as side effects of the ECT treatment, the reason for ECT referral and the previous AP used.

In conclusion, this retrospective study found a high use of ECT among hospitalized adolescent patients with schizophrenia in a major tertiary hospital in China. Although highly treatment resistant nature of patients and the severity of illness in this hospital may help explain the high rate of ECT, reasons for the high use of ECT for adolescent patients in this hospital should warrant urgent investigations.

## Abbreviations

ECT: electroconvulsive therapy
ECMS: electronic chart management system
AACAP: The American Academy of Child and Adolescent Psychiatry
SZ: schizophrenia
MDD: major depression disorder
BD: bipolar disorder
SGAs: second-generation antipsychotics
FGAs: first-generation antipsychotics
